# Multi-Response Optimization of Electrical Discharge Drilling Process of SS304 for Energy Efficiency, Product Quality, and Productivity

**DOI:** 10.3390/ma13132897

**Published:** 2020-06-28

**Authors:** Trung-Thanh Nguyen, Van-Tuan Tran, Mozammel Mia

**Affiliations:** 1 Institute of Research and Development, Duy Tan University, 03 Quang Trung, Da Nang 550000, Vietnam; trungthanhk21@mta.edu.vn; 2 Department of Manufacturing Technology, Le Quy Don Technical University, 236 Hoang Quoc Viet, Ha Noi 100000, Vietnam; 3 Faculty of Mechanical Engineering, Industrial College, Thanh Vinh, Phu Tho Town, Phu Tho 35000, Vietnam; trantuanmta2012@gmail.com; 4 Department of Mechanical Engineering, Imperial College London, Exhibition Road, South Kensington, London SW7 2AZ, UK

**Keywords:** electrical discharge drilling, energy consumption, expansion of hole, material removal rate, optimization

## Abstract

The electrical discharge drilling (EDD) process is an effective machining approach to produce various holes in difficult-to-cut materials. However, the energy efficiency of the EDD operation has not thoroughly been considered in published works. The aim of the current work is to optimize varied parameters for enhancing the material removal rate (MRR), saving drilled energy (ED), and decreasing the expansion of the hole (HE) for the EDD process. Three advanced factors, including the gap voltage adjustor (GAP), capacitance parallel connection (CAP), and servo sensitivity selection (SV), are considered. The predictive models of the performances were proposed with the support of the adaptive neuro-based fuzzy inference system (ANFIS). An integrative approach combining the analytic hierarchy process (AHP) and the neighborhood cultivation genetic algorithm (NCGA) was employed to select optimal factors. The findings revealed the optimal values of the CAP, GAP, and SV were 6, 5, and 4, respectively. Moreover, the ED and HE were decreased by 16.78% and 28.68%, while the MRR was enhanced by 89.72%, respectively, as compared to the common setting values. The explored outcome can be employed as a technical solution to enhance the energy efficiency, drilled quality, and productivity of the EDD operation.

## 1. Introduction

Electrical discharge machining (EDM) operation is an efficient approach to machine difficult-to-cut materials [1]. EDM processes are extensively applied in industrial applications to produce complex shapes and to achieve high precision. For EDM operations, the material is removed from the surface of a conductive specimen with the support of electrical sparks between the electrode and workpiece. Various EDM processes, including the die-sinking EDM, wire-EDM, powder-mixed EDM, micro-EDM, and dry EDM, were appropriately applied to produce small, fragile, and delicate components due to non-machining forces applied [2]. Furthermore, the EDM process is effectively used to machine the components with different dimensional scales. Unfortunately, EDM operations can only be employed on the electrically conductive workpiece. These processes also have low productivity (e.g., material removal rate), high energy consumed, and low machined quality (e.g., high surface roughness). It is difficult to achieve the precision of the machined shape corner due to the tool wear. The electrode consumption is another factor that has a negative impact on production costs.

The principle of the EDD process is familiar with that of the EDM method. It is a thermal-electric process, which uses a series of discrete sparks to remove the material from the electrically conductive workpiece. The gap between the electrode and the specimen is generated in the dielectric. The dielectric is ionized to form an electric bridge on gaps, which produces the electrical spark. For the EDD process, the long electrode with the internal dielectric fluid rotates around the spindle center. The metal is quickly expelled as the series of ionized dielectric vapor collapse. Electrode rotation provides better flushing, which significantly contributes to the uniformity of the hole profile. It can be stated that the EDD operation is developed to drill holes quickly in the production of turbine blades, coolant lines, ejector holes, mold vent holes, and other industrial applications.

During blind hole drilling, the material is molten and ejected due to electrode rotation. The debris particles are cooled down and accumulated at the bottom surface of the tool. The heap of accumulated debris is produced at the hole bottom due to the vortex motion. The debris is removed in the processing time with the support of a high pressure flow.

Many researchers have previously performed parameter-based optimizations for enhancing the technical responses of the EDD processes. A model of the wear of electrodes was developed for the EDD operation of the die material [3]. The outcomes indicated that the wear amount can be precisely forecast. Yildiz et al. [4] developed the empirical correlation of the depth of the recast layer regarding the applied current (AC), pulse on time (T_on_), and pulse off time (T_off_) for beryllium copper. The authors stated that the regression model had acceptable accuracy. The second-order models of the roughness (R), circularity (CIR), and machining rate (MR) were developed for the EDD process of the super alloy [5]. The optimal values helped in the improvements in MRR, R, and CIR were 82.3%, 39.3%, and 25.0%, respectively. An autonomous system was developed to achieve the optimal factors for minimizing the drilling time (DT), tool wear (TW), and R [6]. The neuro-fuzzy inference technique was utilized to depict the relations between the processing inputs and technological outputs. A new EDD process with the diamond electrode was developed to minimize the R and CIR of the drilled nickel alloy [7]. The significant enhancements of the R and CIR are around 25% and 29%, as compared to the traditional one. The optimized parameters were determined to reduce the R and DT of the EDD operation of nickel alloy [8]. The findings revealed that the AC was found to be the most affected factor.

The process inputs, including the AC, T_on_, tool speed (TS), flow rate (FR), and pressure (P) on the MRR, R, and overcut (OC) for the EDD process were analyzed by Yadav et al. [9]. The explored outcomes revealed that the low cracks, debris, and craters could be achieved using the near-dry EDD implementation. A dry EDD process was proposed to enhance the productivity of the machined characteristics of the composite material [10]. The author stated that the MRR produced by dry EDD could be improved by 200%, as compared to the oil-based one. The oxygen-based EDD depicted better efficiency than the air-based EDD. The impacts of the geometry of the electrode, tool speed (TS), voltage (VO), and capacitance (CA) on the MRR, OC, and tapper angle (TAP) for the EDD process of the stainless steel 316 were analyzed [11]. The authors stated that the CA was found to be the most effective input, followed by the polarity, the TS, and VO. The optimal values of the polarity, TS, VO, and CA were the positive shape, 250 RPM, 100 V, and 0.01 nf. The correlated models of the MR, TW, OC, and TAP were proposed regarding the AC, TS, and the P with the aid of the artificial neural network [12]. The author stated that the AC was the most affecting factor and the proposed model could provide the minimal error. The optimum factors of the AC, TS, and the P were 10.18 A, 100 RPM, and 58.78 bar.

The effects of the process inputs, including the T_on_, VO, and ultrasonic vibration on the recast layer thickness (TL), micro-hardness (MH), and the chemical composition of the micro-holes for the EDM process have been analyzed [13]. The findings revealed that the depth value of the recast layer is in the range of 9–23 μm. The micro-hardness value is in the range of 166–200 HV. The machined surface contains lower amounts of carbon, oxygen, and tungsten. A novel turning spindle was developed to produce the free-form cylindrical geometries on SS304 specimens [14]. The impacts of the T_on_, AC, VO, and tool thickness (TN) on the MRR and TW were investigated. The authors stated that the T_on_ was the most effective factor that affected the MRR and TW, followed by the VO, TN, and AC, respectively. The second-order model of the roughness in terms of the T_on_, T_off_, and AC was developed for the EDM process of SS304 [15]. Moreover, the authors stated that the AC and T_on_ were significant factors. The influence of the polarity on the TW and MRR for the micro EDM process of SS304 was explored by Boban et al. [16]. The outcomes revealed that the direct polarity has an effective contribution to a reduction in the TW and maximization of MRR.

Pandey et al. [17] used a hybrid algorithm to optimize the T_on_, T_off_, P, and AC for minimizing the CIR, TAP, and dilation (DI) of the drilled nickel alloy. The outcomes revealed that the correlations may be applied for the prediction of EDD responses. The desirable values of the T_on_, T_off_, P, and AC were 8 μs, 7 μs, 60 bar, and 10 Amp, respectively. The advantages of the cryogenic cooling-based EDD operation were explored by Manivannan and Kumar [18]. The authors presented that the enhancements in the MRR and roughness were 62% and 36%. The effectiveness of the varied electrodes on the MRR, TW, OC, R, and aspect ratio (AR) was analyzed in the drilling of AISI 304 [19]. Elliptical shape was considered as the best solution to obtain higher MRR and lower TW. The elliptical geometry may be used to observe less OC, lower R, and higher AR. Ununea et al. [20] revealed that the hole quality (OC and TAP) and productivity (MRR and TW) of the EDD process for Inconel 718 could be improved with the support of a vibration-assisted module. The authors emphasized that the vibration significantly helped to remove the debris and improve the flushing as well as the machining stability. The influences of the different tool materials on the MRR, TW, TAP, OC, and roughness of the drilled SS 316L were analyzed [21]. The outcomes revealed that the tungsten tool contributed to the lowest values of the TWR as well as OC and the MRR could be improved using the copper tool. Moreover, a smooth surface was obtained at low discharge energy. The effects of the T_on_, V, and CA on the dimple responses (depth, height, and width) for the EDD operation of a titanium alloy were analyzed by Jung et al. [22]. The predictive correlations with 95% confidence were designed. The optimum values of the T_on_, V, and CA were 100 μs, 180 V, and 10,000 pF, respectively. The impacts of the AC, T_on_, TS, and duty cycle (DC) on the MR, TW, and OC on the EDD operation of mold material were investigated by Singh et al. [23]. The authors stated that the EDD operation employing the rotary electrode provided a higher MRR and a lower TWR as well as OC.

The regression models were used to address the correlations between inputs (AC, T_on_, T_off_, and P) and the hole quality (CIR, TAP, and expansion) of the drilled titanium [24]. The authors emphasized that the proposed formulas can be accurately used to estimate the response values. The general improvements of the EDD responses are around 59%. A new measurement is proposed to explore the TW of the drilled hole [25]. The error of 1% revealed the effectiveness of the measuring method. The machining program will be updated after each operation to decrease the idle time. The influences of various fluids on the formation of the white layer were analyzed by Li et al. [26]. The author concluded that a higher conductivity of the fluid resulted in a thicker white layer and an increased cooling speed caused a thin affected layer. Moreover, the lowest thickness of the white layer could be obtained with the support of the kerosene fluid. The impacts of various fluids on the MR, TW, and hole properties were experimentally explored for the EDM process of the titanium [27]. The author stated that the mixture of the Cu powder and dielectric fluid significantly enhanced the EDD responses. The influences of the TS, AC, T_on_, and FR, and the quantity of oxygen on the MRR, OC, and surface characteristics were thoroughly explored [28]. The outcomes revealed that the oxygen gas-assisted EDD process has an efficient contribution to improve the MRR and decrease the thicker layer as well as the OC.

The inputs considered are the processing conditions (AC, T_on_, T_off_, and VO), the electrode’s characteristics (geometry and materials), dielectric properties (type, dielectric strength, and viscosity), and the workpiece materials. The technological responses considered are the hole quality (CIR, TAP, DI), surface properties, MRR, and TW. However, the drawbacks of the published works are listed as follows:

The impacts of the advanced parameters, including the gap voltage adjustor (GAP), capacitance parallel connection selecting (CAP), and servo sensitivity selection (SV) on the EDD responses, such as energy consumed, the hole quality, and productivity have not been thoroughly considered. These parameters have efficient impacts on the machining speed, energy stability, and the formation of the hole. The formulations having acceptable accuracy between the advanced parameters and the performances measured for the EDD operations have not been considered in the works. The selection of optimal values of these factors for maximizing the hole quality as well as machining rate and minimal drilled energy has not been considered in the works published. The regression models, such as the linear and second-order formulations were widely applied to depict the relations between the processing inputs and machining characteristics in the EDD optimizations. Unfortunately, these mentioned approaches have low predictive accuracy due to the approximate behavior [29,30]. It is necessary to propose a higher accurate forecasting model for the optimization issues in the EDD process. The Taguchi method and Taguchi-based approaches have been widely used to select the optimal factors in EDD optimization. However, the optimal outcomes selected from the experimental data have a high risk of obtaining the local result, which leads to inefficient optimization. The development of a new optimization method is an urgent demand to guarantee the selection of global results.

This work presents an optimizing approach of the EDD process of the stainless steel 304 (SS304) to minimize the HE and the ED, while MRR is to be maximized. SS304 is a common stainless steel, which is widely applied in industrial applications for automotive, molding, and aerospace components [31]. It is a difficult-to-cut material due to the superior characteristics, including the good corrosion resistance, chemical reaction resistance, low thermal conductivity, and high strength in the high temperature working conditions. The interpolative ANFIS correlations are used to establish relations between the advanced conditions and EDD responses. The NCGA is utilized to generate feasible solutions and determine the best design point.

## 2. Materials and Methods

### 2.1. Optimizing Objectives

**Figure 1:**
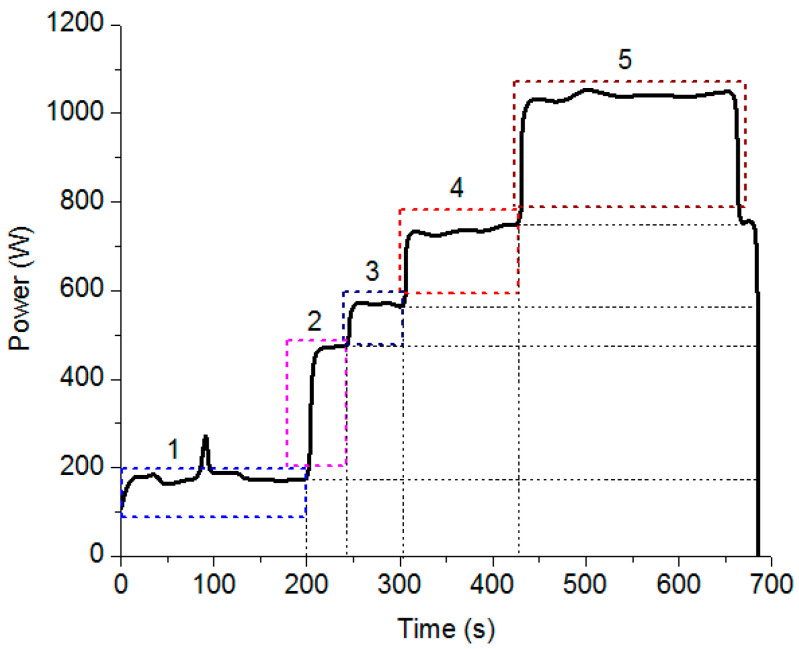
The profile of the power consumption in the EDD process.

The power consumption in the EDD process (P_D_) can be divided into five primary components, as shown in Figure 1:

- Power is consumed for the computer numerical control system (P_CNC_) to control the machine’s components and realize machine–human interaction.
- Power is consumed for the cooling system (P_C_) to supply the machining fluid with the functions of flushing, cooling, and debris-removal.
- Power is consumed for the tool rotational system (P_TRS_) to supply the rotation of the electrode.
- Power is consumed for the tool feeding system (P_TFS_) to supply linear motion in the Z-axis.
- Power is consumed for the pulse system (P_PS_) to supply pulse energy during the drilling time.

Therefore, the total energy consumption of the EDD operation can be calculated by Equation (1).

(1) $$E_{D}=\int_{0}^{t_{\mathrm{cnc}}}P_{\mathrm{CNC}}\mathrm{dt}+\int_{0}^{t_{c}}P_{C}\mathrm{dt}+\int_{0}^{t_{\mathrm{trs}}}P_{\mathrm{TRS}}\mathrm{dt}+\int_{0}^{t_{\mathrm{tfs}}}P_{\mathrm{TFS}}\mathrm{dt}+\int_{0}^{t_{\mathrm{ps}}}P_{\mathrm{PS}}\mathrm{dt}=\int_{0}^{t_{d}}P_{D}\times \mathrm{dt}$$

where P_D_ and t_d_ denote the drilled power and the drilling time, respectively.

**Figure 2:**
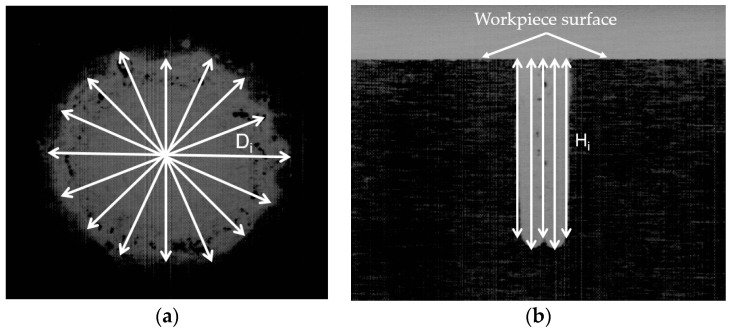
Calculation of the drilled dimensions: (**a**) A schematic calculation of the drilled diameter; (**b**) A schematic calculation of the drilled hole.

The expansion of the hole (HE) is calculated using Equation (2) (Figure 2a).

(2) $$\mathrm{HE}=D_{\mathrm{at}}-D_{e}=\frac{D_{1}+D_{2}+\ldots +D_{n}}{n}-D_{e}$$

where D_at_ and D_e_ are the average measurement of the top diameter of the drilled hole and the electrode’s diameter, respectively. D_i_ and n are the diameter of i_th_ position measured and the number of measuring points.

The drilled MRR value is calculated using Equation (3).

(3) $$\mathrm{MRR}=\frac{\Pi H{\left(D_{\mathrm{at}}+D_{\mathrm{ab}}\right)}^{2}}{16t_{D}}$$

where D_ab_ denotes the average measurement of the bottom diameter. H is the drilled length, which is calculated using Equation (4) (Figure 2b).

(4) $$H=\frac{\sum_{1}^{n}H_{i}}{n}$$

where H_i_ and n are the drilled length of i_th_ position measured and the number of measuring points.

Besides the common factors, such as the T_on_, T_off_, AC, and VO, three advanced parameters, including the gap voltage adjustor (GAP), capacitance parallel connection (CAP), and servo sensitivity (SV) have significantly affected the power consumed, drilling time, and drilled quality. Practically, the higher GAP is, the lower the drilling time will be. However, an increased GAP may cause difficulty in a draining job. The drilling speed can be adjusted using the variety of the CAP value. The bigger the CAP is, the lower the machining time will be, but the higher the wear of the electrode. Moreover, the EDD process requires more drilling time without the CAP. The speed of the Z-axis can be controlled by the SV value. The bigger the SV, the faster the Z-axis will be. The SV influences the applied voltage and energy stability. Consequently, it is necessary to select the appropriate values of three advanced parameters. The ranges of the GAP, CAP, and SV are shown in Table 1. The factor’s levels are primarily identified based on the guidance of the manual book for the EDD machine.

A multi-objective optimization can be presented as:

Finding the optimal values of CAP, GAP, and SV.
Maximize MRR; Minimize ED and HE.
Constraints: 1 ≤ CAP ≤ 9; 2 ≤ GAP ≤ 8; 2 ≤ SV ≤ 8.

### 2.2. Optimization Procedure

**Figure 3:**
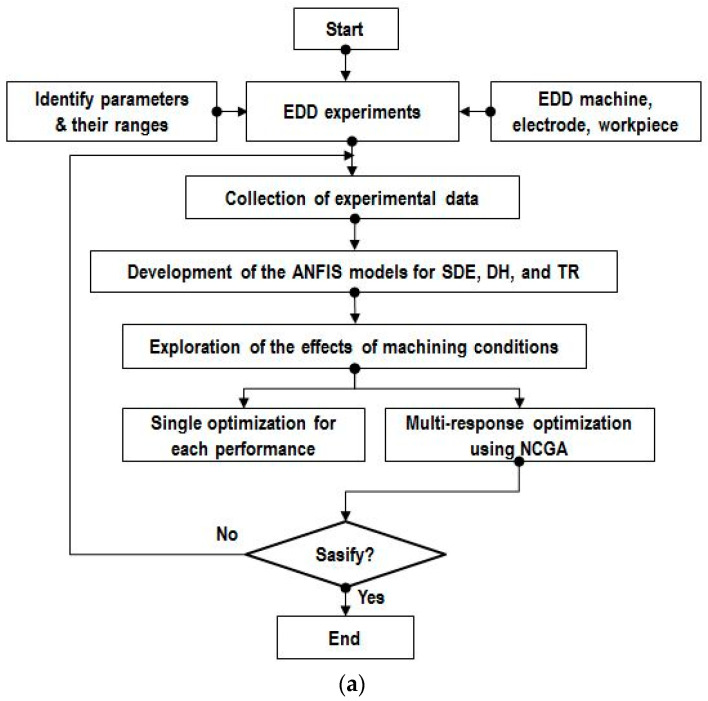

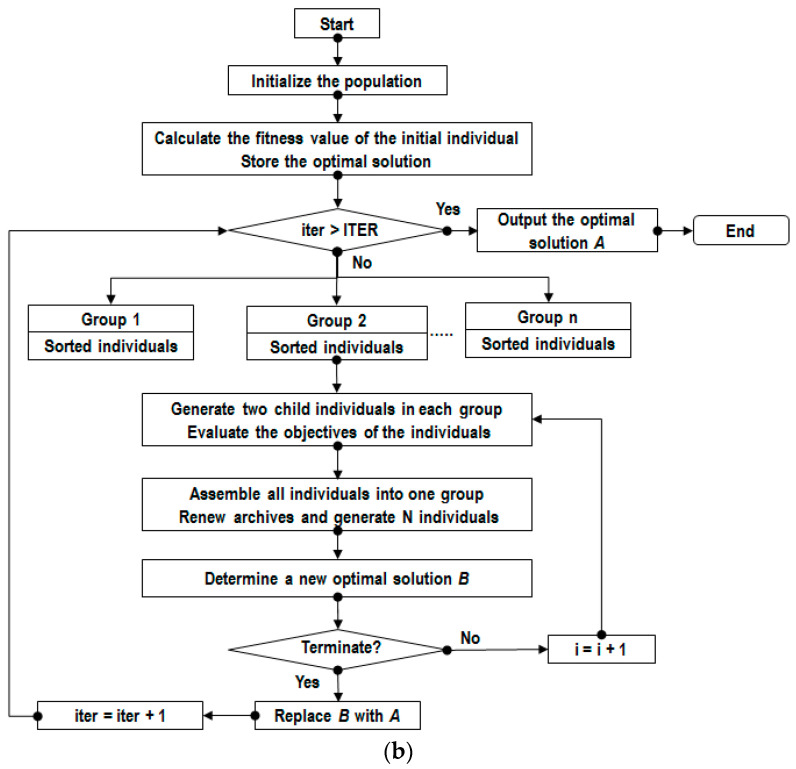
Optimization approach: (**a**) Systematic optimization procedure; (**b**) The operating procedure of the NCGA.

The optimization procedure for generating optimal values is shown in Figure 3a.

Step 1: The EDD trials using full experimental design are conducted to collect the necessary data [32,33].

Step 2: The predictive models of the ED, HE, and MRR are then constructed in terms of the advanced parameters using the ANFIS. The ANFIS model is a powerful approach to depict the highly nonlinear data [34]. The advantages of the artificial neural network and fuzzy system are integrated into the ANFIS model [35]. The inputs are transformed into the desired outputs based on the interconnection of fuzzy rules and process elements of the neural network. The development of ANFIS models can be divided into two operating stages, including training and testing operations. Firstly, the ANFIS network of each response is constructed in the training stage. The accuracy of the trained model is then validated using testing data. The number of membership functions, different types of membership functions (MF), and learning algorithms are used to achieve minimal errors.

The adequacy of the developed ANFIS model can be assessed using the coefficient of determination (R^2^ value), which is calculated using Equation (5).

(5) $$R^{2}=1-\frac{\sum_{i=1}^{n}{\left(y_{i}-{\hat{y}}_{i}\right)}^{2}}{\sum_{i=1}^{n}{\left(y_{i}-{\bar{y}}_{i}\right)}^{2}}$$

where $y_{i},\;{\;\bar{y}}_{i}$ and ${\;\hat{y}}_{i}$ present the experimental value, the mean of obtained value, and the approximated value, respectively.

Step 3: Determine the weight values of the process responses using AHP [36].

In this work, we consider three EDD responses including the ED, HE, and MRR. A pairwise matrix is constructed using the nine-point Saaty scale (Table 2). The matrix element (a_ij_) is assigned based on the value of relative importance. The a_ij_ of 1 is applied when the attribute compares with itself. The reciprocated value (a_ji_) is assigned to the corresponding element (a_ji_ = 1/a_ij_).

The values of the geometric mean (GM) and normalized weights are calculated using Equations (6) and (7).

(6) $$\mathrm{GM}={\left[\overset{m}{\underset{j=1}{\prod }}a_{\mathrm{ij}}\right]}^{1/m}$$

(7) $$w_{j}=\frac{{\mathrm{GM}}_{i}}{\sum_{i=1}^{m}{\mathrm{GM}}_{i}}$$

where m denotes the number of attributes.

The values of the consistency index (CI) and consistency ratio (CR) are computed using Equations (8) and (9).

(8) $$\mathrm{CI}=\frac{\lambda_{\max-m}}{m-1}$$

(9) $$\mathrm{CR}=\frac{\mathrm{CI}}{\mathrm{RI}}$$

where λ_max_ and RI are the estimated value and random index, respectively. The RI value depends on number of attributes (Table 3). The CI value reflects the consistency of the proposed method. In contrast, the judgment is necessary to consider when the CI value does not satisfy the requirement. The CR value of 0.1 or less is preferred, which reveals the consistency of the assigned pairwise comparison matrix.

Step 4: An evolutionary algorithm called the neighborhood cultivation genetic algorithm (NCGA) is used to select the optimal factors, in which a neighborhood crossover operation is added into the mechanisms of the algorithm. The population is divided into groups that consist of two together individuals. In NCGA, most of the genetic operations are performed in a group that consists of two individuals. The NCGA is an efficient technique, which is well suited to solve highly nonlinear problems with minimal running time, as compared to the traditional ones, such as NSGA-II and SPEA2. The operating algorithm of the NCGA is listed as below (Figure 3b):

- The start population (P_0_) is generated and the fitness value of the initial individual is calculated. The P_t_ is copied into A_0_ and the archive size (N) is produced.
- A new population (P_t_) of the new generation is generated.
- The individuals of the P_t_ are sorted according to the purpose of the objective. The aimed response is sequentially changed at every generation.
- P_t_ is divided into groups that consist of two individuals. These two individuals are chosen from the top to the bottom of the sorted individuals.
- In a group, the crossover and mutation operations are performed. In these processes, two-child individuals are generated, and the parent individuals are eliminated.
- All the objectives of individuals are derived.
- All individuals are assembled into the same group and a new population (P_t_) is produced.
- The P_t_ is assembled with the A_t-1_ and N individuals are selected.
- If the terminal condition is satisfied, the procedure is terminated. In contrast, the simulation returns to step 2.

### 2.3. Experiments and Measurements

**Figure 4:**
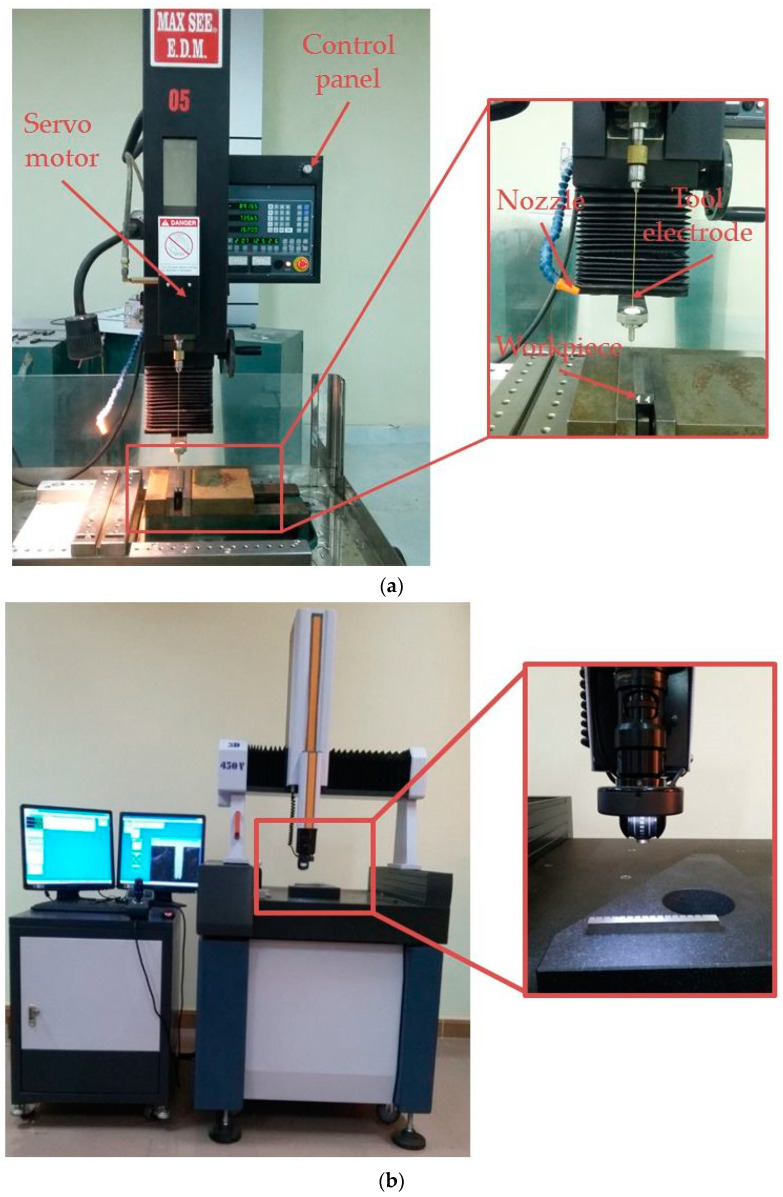
The test stand: (**a**) EDD experimental setup; (**b**) Measuring dimension.

The CNC EDD machine entitled MAX SEE S36 (DYNARCO SDN BHD, Penang, Malaysia) is employed to perform the experiments (Figure 4). The dimensions of the workpiece prepared are 20 × 10 × 100 mm^3^. The specimen’s surfaces are ground and polished before the EDD operations. Two of the workpiece’s parts are assembled by means of the mating interface to estimate the drilled length. The workpiece is fixed on the machine’s table using the precision vector vise. Table 4 illustrates the chemical compositions of SS304.

The copper tubular electrode with the single channel is used to perform the EDD trials. The total length, outside diameter, and inside diameter are 400, 1, and 0.36 mm, respectively. The hole center flushing technique is employed to remove the debris particles from the machined specimen. Pure water is applied as a dielectric fluid during the EDD experiment. Dielectric fluid is pumped to the drilling region through the interior channel of the electrode. The drilling program is stopped when the setting depth reaches to 10 mm for each run.

The EDD trials at the highest and lowest values are conducted to ensure the machinability of the specimens. The fixed values of the T_on_, T_off_, AC, VO, and water pressure are 4 μs, 10 μs, 7 A, 60 V, and 75 kg/cm^2^, respectively.

**Figure 5:**
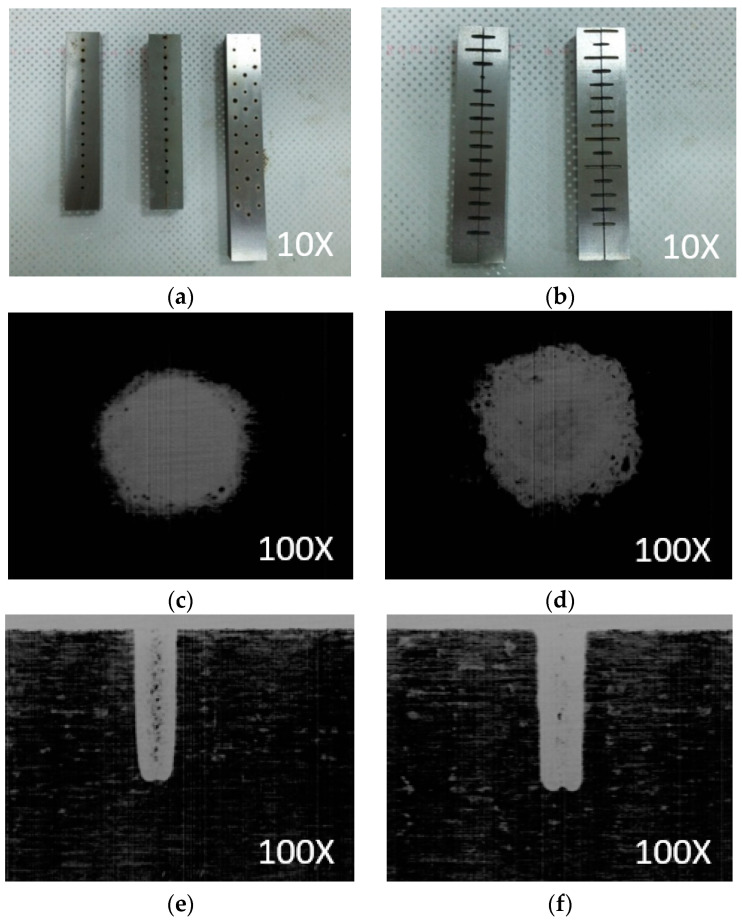
The representative values of the EDD responses: (**a**) Top view of the machined specimen; (**b**) The separated parts of machined specimen; (**c**) The drilled diameter at GAP = 5, CAP = 1, and SV = 2; (**d**) The drilled diameter at GAP = 5, CAP = 9, and SV = 2; (**e**) The drilled length at GAP = 2, CAP = 1, and SV = 5; (**f**) The drilled length at GAP = 2, CAP = 5, and SV = 2.

The power consumption is automatically recorded using a power meter. A high resolution scanner is used to capture and investigate the dimensions of the drilled specimen. The top and side views of the drilled specimen are depicted in Figure 5a,b. The scanned diameters are shown in Figure 5c,d. The scanned lengths are illustrated in Figure 5e,f.

As shown in Figure 5c,d, the errors of the machined hole, such as deterioration and roundness, are produced due to the formation of the recast layer at the rim of the hole. The phenomena can be explained as follows. Higher value of the CAP leads to higher discharge energy, which causes violent sparks; hence, deeper erosion on the surface is produced. After the cooling process, residues remained at the periphery of the hole.

As shown in Figure 5c,d, the hole taper is produced at the EDD experiments. The phenomena can be expressed as follows. The heat generation in the gap between the electrode and workpiece causes intensive discharges. Large amounts of the molten and floating metal are suspended. The tool and specimen are simultaneously deteriorated. When the tool protrudes into a workpiece, the sparking area and conditions continuously change. The wear of the electrode is observed in both bottom and lateral faces. The tool wear deteriorates both the depth and the shape of the machined hole. The corner wear of the electrode will also cause the edge rounding of the machined hole.

The vortex motion causes a heap of the debris particles at the hole bottom. The intensive discharges between the accumulated debris and the tool eventually remove the material from the electrode. A concave tool tip and a protrusion at the hole bottom are produced.

## 3. Results and Discussion

### 3.1. Development of ANFIS Models

Thirty-three experiments were done based on a full factorial experimental design. The experimental result of the EDD process is shown in Table 5. The explored data from 1 to 27 were used to design the ANFIS models. The varied data from 28 to 33 were adopted to check the accuracy of the ANFIS models.

**Figure 6:**
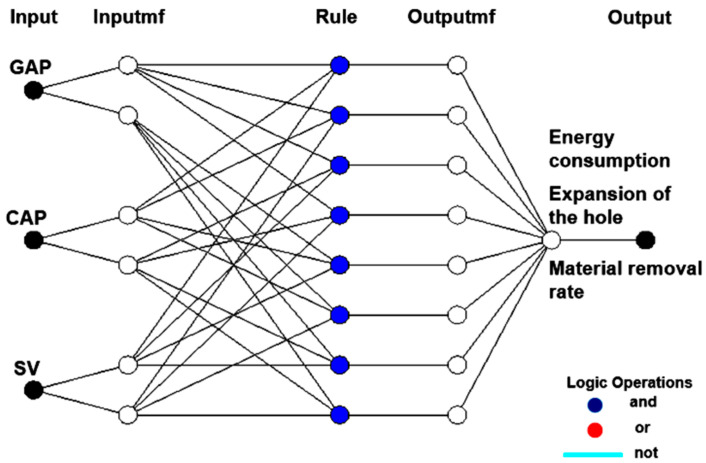
Structure of proposed ANFIS models for the EDD responses.

The ANFIS architecture with three inputs and three outputs is shown in Figure 6. The values used of the epochs and the number of rules are 200 and 32, respectively. Table 6 shows the trained results of the 2-2-2 structures of ANFIS models using different memberships. It can be stated that the lowest value of the global error was obtained using the *gaussmf* membership function. The R^2^ values of the ANFIS models of the ED, HE, and MRR are 0.9726, 0.9787, and 0.9735, respectively, indicating the competitiveness of the proposed correlations.

**Figure 7:**
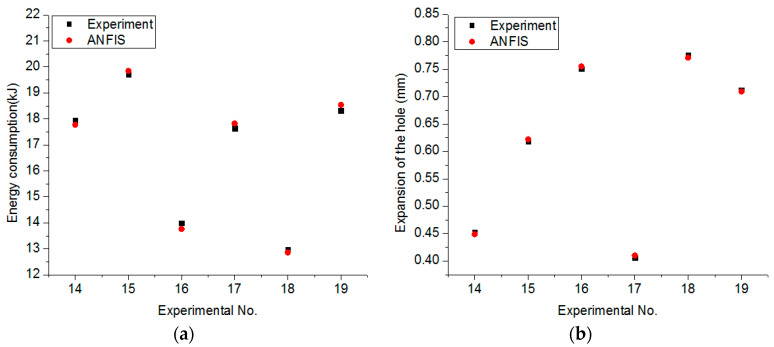

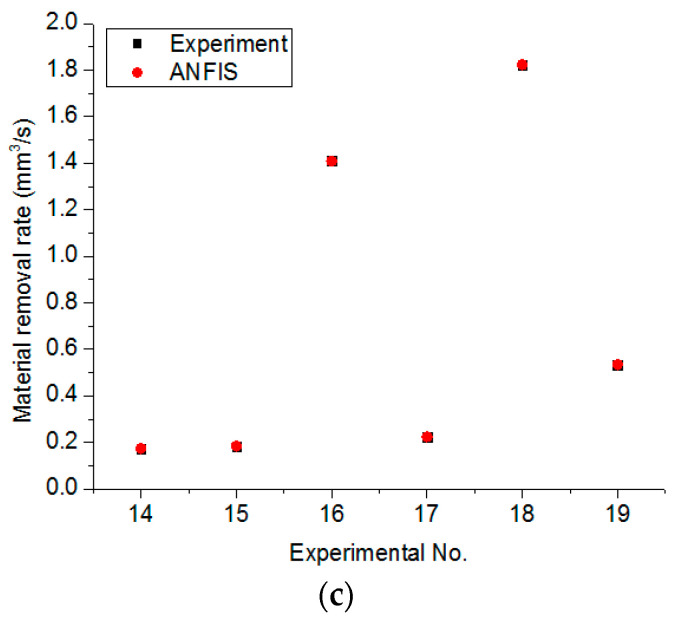
Comparisons between the ANFIS and experimental values: (**a**) For the energy consumed model; (**b**) For the expansion of the hole model; (**c**) For the material removal rate model.

Figure 7 depicts the comparisons between the predicted and experimental values at the random points. It can be stated that high consistency is obtained, indicating an acceptable accuracy of the proposed ANFIS models.

### 3.2. Parametric Impacts

**Figure 8:**
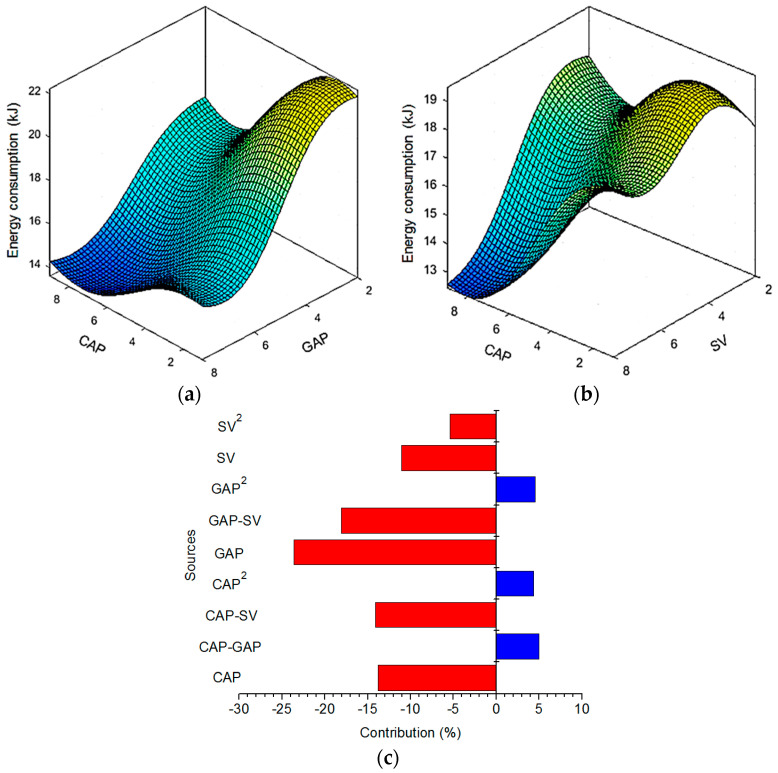
Interaction effects of machining parameters on the ED: (**a**) ED versus CAP and GAP; (**b**) ED versus CAP and SV; (**c**) Parametric contributions of the varied factors.

The drilled energy (ED) is a good indicator to evaluate the environmental influence of the EDD process. In the EDD process, the ED is expressed as the variety in the power used under the effects of the varied inputs. Lower ED is desirable. The influences of the process inputs on the ED are depicted in Figure 8.

As depicted in Figure 8a, a low ED is found with an increased CAP and/or GAP. When applied GAP increases, higher power is consumed, resulting in a more intense spark. The heat flux available for the melting is more, thus, more material is removed from the cutting front. Obviously, more material is easily processed, and a lower drilling time is obtained. As the CAP increases, higher drilling power is consumed, leading to a higher electrical spark. Larger amounts of material are processed and evaporated, which significantly contributes to a reduction in the drilling time. In other words, a higher machining speed is obtained. It can be stated that higher CAP and/or GAP leads to low energy consumed due to lower drilling time.

Figure 8b revealed that an increment in the SV significantly contributes to a decreased ED. The speed of the Z-axis is adjusted using the SV. The higher SV is the faster motion of the Z-axis. As the SV increases, the power used in the feed drive system increases. Fortunately, higher SV leads to a reduction in the drilling time; hence, low drilling energy is obtained.

The parametric contributions of the varied factors for the ED model are shown in Figure 8c. The blue and red colors present the positive and negative impacts. The GAP has the highest contribution (23.62%) in terms of single impact, followed by the CAP (13.81%) and SV (11.05%), respectively. The parametric contributions of the SV^2^, GAP^2^, and CAP^2^ are 5.38%, 4.58%, and 4.36%, respectively. Moreover, for the interaction effects, the contributions of the GAP–SV, CAP–SV, and CAP–GAP are 18.09%, 14.10%, and 5.01%, respectively.

**Figure 9:**
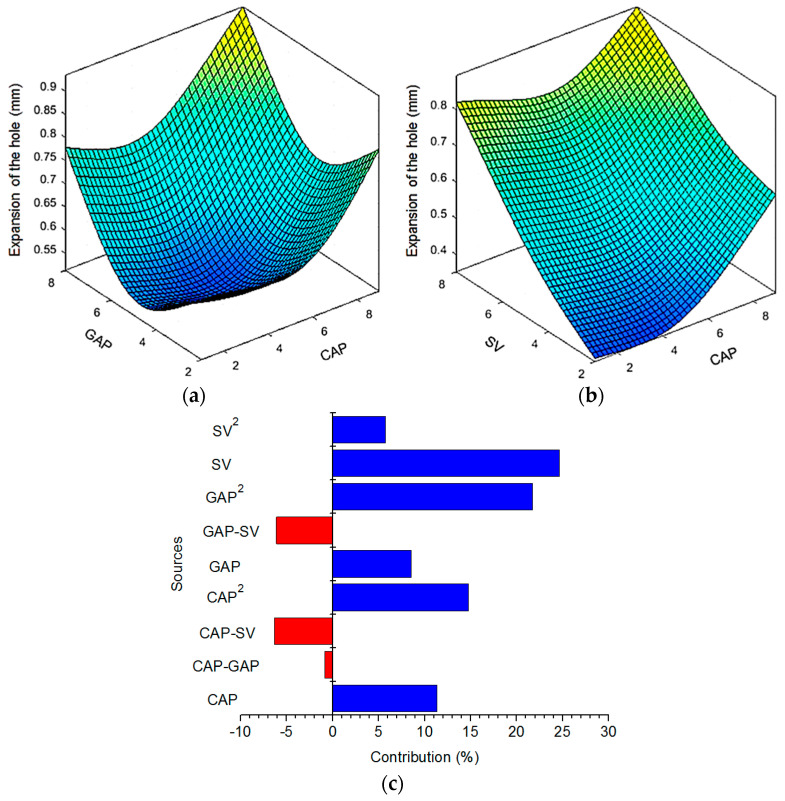
Interaction effects of machining parameters on the HE: (**a**) HE versus CAP and GAP; (**b**) HE versus CAP and SV; (**c**) Parametric contributions of the varied factors.

In the EDD process, the HE is expressed as the changes in the resulting diameter under the effects of the process inputs. A low expansion of the hole is preferred and a HE of zero is the desired purpose. The effects of the varied parameters (GAP, CAP, SV) on the HE is depicted in Figure 9.

It can be stated that the expansion of the hole decreases when the GAP and CAP are on the rise (Figure 9a). After the minimal point, the increased expansion is produced with an increment in GAP or CAP. An increased GAP or CAP leads to higher discharge energy and more debris production. The debris may be located on the drilled surface, which increases the erosion of the electrode. The diameter of the tool is decreased, and a smaller diameter is produced. A further increment in the GAP or CAP causes a higher spark and more material is melted and vaporized; hence, a higher expansion is produced. Moreover, the debris is effectively flushed out at a high value of GAP and/or CAP, which causes an increase in the drilled diameter.

According to Figure 9b, selection of a high servo sensitivity leads to an increment in the HE. A low discharge spark is obtained at a low SV; hence, a small amount of material is vaporized. As a result, a small diameter is generated. A higher SV leads to an increment in the electrical spark and an uneven distribution of discharge energy. Consequently, a larger diameter is produced. To increase the hole quality, the value of the SV should be kept lower, while the middle levels of the GAP and CAP are recommended to use.

Figure 9c presents the contributions of the varied parameters for the HE models. For the single impact, the contributions of the SV, CAP, and GAP are 24.63%, 11.33%, and 8.54%, respectively. For the quadratic impact, the parametric contributions of the GAP^2^, CAP^2^, and SV^2^ are 21.73%, 14.78%, and 5.74%, respectively. For the interaction effects, the contributions of the CAP–SV, GAP–SV, and CAP–GAP are 6.31%, 6.11%, and 0.84%, respectively.

**Figure 10:**
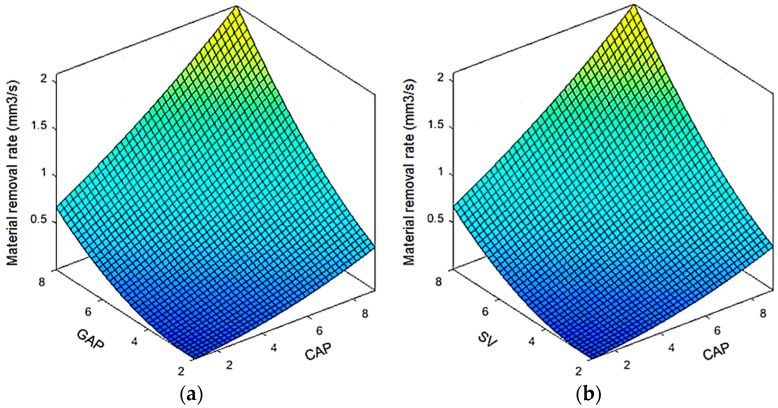

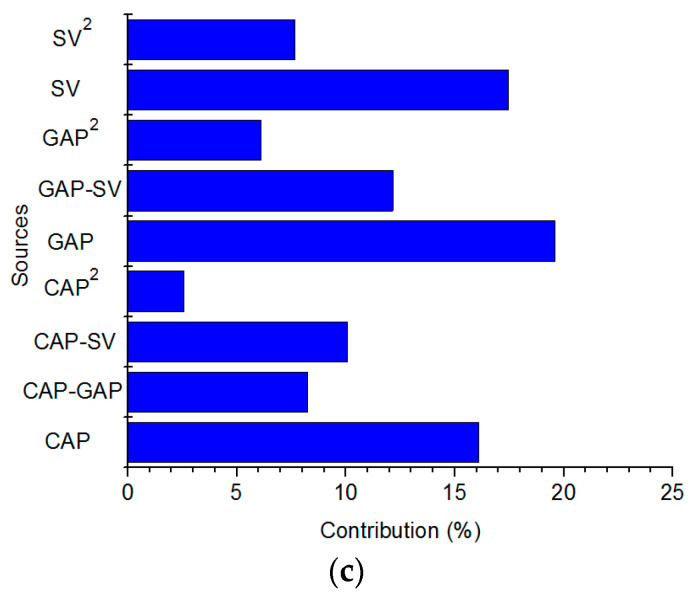
Interaction effects of machining parameters on the MRR: (**a**) MRR versus CAP and GAP; (**b**) MRR versus CAP and SV; (**c**) Parametric contributions of the varied factors.

In the EDD operations, higher MRR values are preferred to increase the drilling efficiency. The influences of the varied inputs on the MRR are shown in Figure 10. Generally, the MRR is enhanced with an increased GAP, CAP, and SV. With an increase in the input from the lowest and highest values, the MRR impressively increases. Figure 10a indicates that the MRR values increase in parallel to an increased GAP. When a higher GAP applies, the increased electrical spark causes more melted and evaporated material. As a result, a larger dimension is produced on the workpiece.

At a higher value of the CAP, a higher discharge energy is generated; hence, more material is melted and evaporated. Therefore, a higher amount of material is processed, and the machining productivity is enhanced. The similar effect of the SV on the MRR can be found in Figure 10b.

The parametric contributions of the varied parameters for the HE model are shown in Figure 10c. For the single impact, the GAP has the highest contribution (19.62%), followed by the SV (17.44%) and SV (16.10%), respectively. The parametric contributions of the SV^2^, GAP^2^, and CAP^2^ are 7.68%, 6.12%, and 2.59%, respectively. For the interaction effects, the contributions of the GAP–SV, CAP–SV, and CAP–GAP are 12.16%, 10.06%, and 8.23%, respectively.

**Figure 11:**
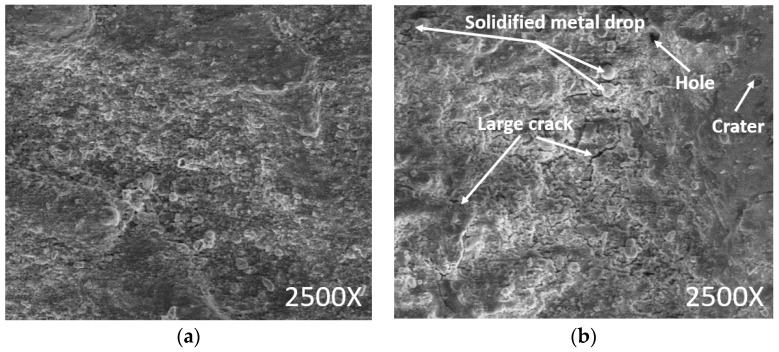
Drilled surface at the various inputs: (**a**) Drilled surface at the GAP = 2, CAP = 1, and SV = 5; (**b**) Drilled surface at the GAP = 8, CAP = 1, and SV = 5.

The EDD surfaces at different experimental conditions are shown in Figure 11. A smoother surface with small cracks and holes is depicted at a low value of the GAP (Figure 11a). The melted metal is converted into small globules. However, a coarser surface with bigger craters, cracks, and holes is produced at a higher value of the GAP (Figure 11b).

### 3.3. Generation of Optimization Results

In the current work, single and multi-objective optimizations are simultaneously performed. The proposed models can be adopted to obtain the optimal inputs for improvements in energy efficiency, hole quality, and machining rate.

Table 7 exhibits the optimizing results of three scenarios for every single optimization. For minimizing energy drilled, the optimal values of the CAP, GAP, and SV are 9, 8, and 8, respectively, as shown in the first scenario. The second case presents the optimal values of the CAP (3), GAP (4), and SV (2) to minimize the expansion. The third scenario aims to achieve the maximum MRR, in which the optimum values of the CAP, GAP, and SV are 9, 8, and 8, respectively.

For multi-response optimization, the weights of the EDD performances are necessary to select. The objective of this work is to enhance productivity as well as quality and decrease energy consumption of the EDD process of SS304. Thus, the material removal rate is assigned at the maximum weight. The expansion of the hole is placed on the second number and drilled energy is given the least importance. The values of the correlated pairwise for the EDD responses are shown in Table 8.

The calculated weights are used avoid the subjective judgment of the operator and exhibit the objective impact of the varied factors on the EDD performances. The weights are satisfied by the rule of Equation (10).

(10) $$\overset{m}{\underset{j=1}{\sum }}\omega_{j}=1$$

The values of the geometric mean and weights are listed in Table 9. The values of the CI and CR are 0.0295 and 0.0567, respectively. A CR value less than 0.1 indicates the consistency of the AHP method. Therefore, the weight values of the ED, MRR, and HE are 0.19, 0.66, and 0.15, respectively.

**Figure 12:**
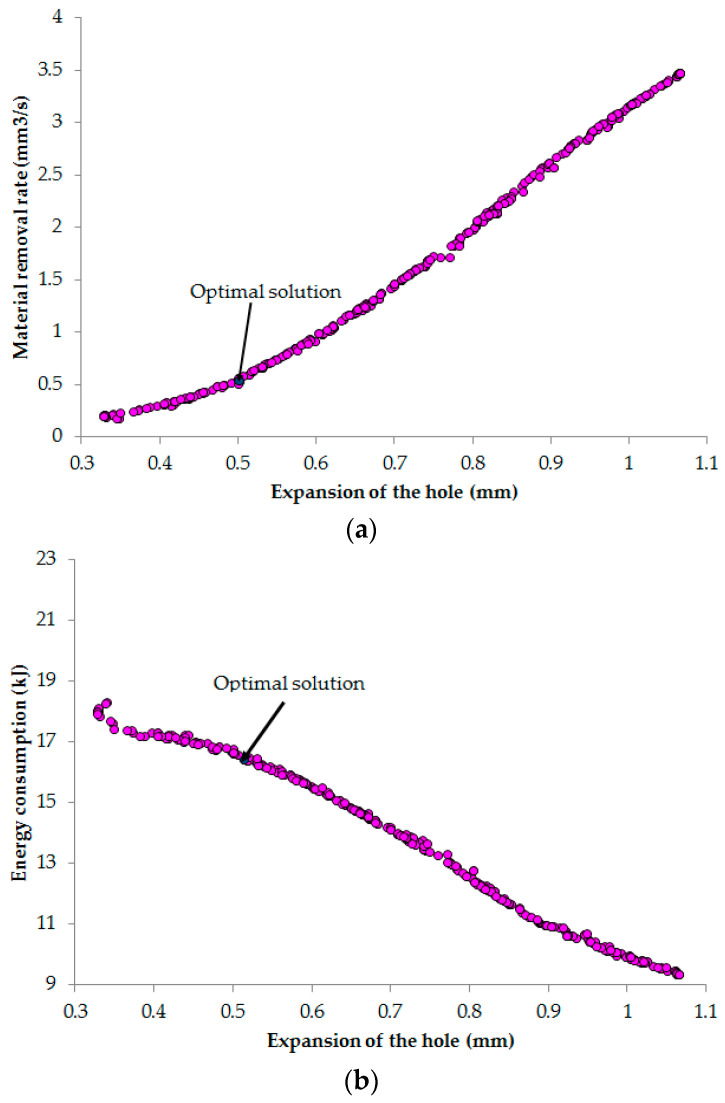
Pareto fronts: (**a**) MRR versus HE; (**b**) ED versus HE.

To solve the multi-objective optimization that minimizes ED as well as HE and maximizes MRR, the NCGA was utilized to predict the optimal process parameters. Table 10 presents the setting parameter of the NCGA algorithm. The iteration of 800 is employed to obtain the convergence. The Pareto fronts (Pareto optimal set) generated by NCGA are depicted in Figure 12. It can be stated that it is unfeasible to achieve minimum ED as well as HE and maximum MRR simultaneously because the objectives are conflicted. If we want to increase MRR or decrease ED, the expansion of the hole (HE) also increases. When MRR decreases or ED increases, the hole quality significantly improves. A representative scenario having optimal values of the inputs and outputs is shown in Table 11. In this case, the MRR is enhanced by 89.72% and the reductions in the ED and HE are 16.78% and 28.68%, respectively, as compared to the common values used.

To investigate the accuracy of the applied approach, a confirmatory experiment is performed at an optimal solution. The comparison findings are shown in Table 12. The prediction errors of the ED, HE, and MRR are 1.55%, 2.60%, and 1.58%, respectively. The small values of the errors reveal a good correlation between the optimized and experimental data. The similar findings of the prediction errors were presented in the research published [10,24]. Consequently, the precision of the developed approach is acceptable for the optimization of the EDD processes.

## 4. Conclusions

The current research addressed the optimization of EDD for the material SS304 with the objectives of decreasing the expansion of the hole (HE) as well as the drilled energy (ED) and improving the material removal rate (MRR). The predictive models between advanced process parameters and EDD responses were constructed with the support of the ANFIS models. The NCGA and Pareto graphs were applied to derive out the optimum values of the factors considered. Some conclusions obtained from this work can be drawn as follows:

- The ANFIS models have been constructed for different EDD characteristics. The statistical outcomes indicate that the established models for the EDD responses are sound and reliable. The ANFIS models of the EDD performances have shown a high precision for predictive purposes. The modeling technique can be effectively employed to depict the highly nonlinear relations of the EDD performances.
- All advanced process parameters, including the gap voltage adjustor (GAP), capacitance parallel connection (CAP), and servo sensitivity (SV) have significant impacts on the EDD characteristics. Higher values of the GAP, CAP, and SV should be selected to increase the MRR and decrease the ED. The lowest level of the SV can be applied to achieve low expansion. The middle levels of the GAP and CAP are recommended to enhance the drilled quality.
- The optimal factor setting obtained by NCGA of the CAP, GAP, and SV are 6, 5, and 4, respectively. As compared to random values, the ED and HE are decreased by 16.78% and 28.68%, respectively. The MRR is improved by around 89.72%.
- The hybrid approach including the ANFIS model and NCGA can extensively support the optimization of the EDD process, in which objectives are generally conflicting with each other. Moreover, the obtained finding is an efficient technique to select the optimal varied parameters for the machine operator.

This work addressed three EDD responses including the drilled energy, the expansion of the hole, and the material removal rate that were considered as objective outputs. Other responses such as over-cut and tool wear rate can be studied to holistically optimize the EDD process. Moreover, additional parameters including the residual stress and machining costs can be analyzed in future works.

## Figures and Tables

**Table 1:** Varied parameters and their levels.

Symbol	Parameters	Level 1	Level 2	Level 3
CAP	Capacitance parallel connection	1	5	9
GAP	Gap voltage adjustor	2	5	8
SV	Servo sensitivity selection	2	5	8

**Table 2:** Saaty’s preference scale [37].

Judgment of Preference	Numerical Rating
Extremely preferred	9
Very strongly to extremely preferred	8
Very strongly preferred	7
Strongly to very strongly preferred	6
Strongly preferred	5
Moderately to strongly preferred	4
Moderately preferred	3
Equally to moderately preferred	2
Equally preferred	1

**Table 3:** The values of the RI [38].

N	1	2	3	4	5	6	7	8	9	10
RI	0	0	0.52	0.89	1.11	1.25	1.35	1.4	1.45	1.49

**Table 4:** Chemical compositions of SS304.

C (%)	Mn (%)	P (%)	S (%)	Si (%)	Cr (%)	Ni (%)	Al (%)
0.08	2.00	0.045	0.03	0.75	19.00	10.00	0.10

**Table 5:** Experimental results.

No.	CAP	GAP	SV	P_D_ (W)	ED (kJ)	HE (mm)	MRR (mm^3^/s)
	Obtained data for development of ANFIS models						
1	1	2	2	506.42	19.521	0.407	0.37005
2	1	2	5	563.30	21.433	0.639	0.00665
3	1	2	8	725.59	23.246	0.956	0.09111
4	1	5	2	521.40	17.576	0.361	0.16905
5	1	5	5	658.79	18.537	0.548	0.16026
6	1	5	8	830.78	18.498	0.820	0.59935
7	1	8	2	687.48	17.067	0.637	0.32498
8	1	8	5	827.13	16.278	0.778	0.67080
9	1	8	8	1020.99	13.988	1.005	1.46451
10	5	2	2	526.20	18.092	0.435	0.23084
11	5	2	5	675.14	19.362	0.620	0.16090
12	5	2	8	864.03	19.233	0.890	0.53884
13	5	5	2	663.46	17.259	0.382	0.26975
14	5	5	5	809.60	16.678	0.522	0.55444
15	5	5	8	1010.74	14.697	0.747	1.28699
16	5	8	2	886.34	17.162	0.652	0.66559
17	5	8	5	1028.29	14.531	0.747	1.30489
18	5	8	8	1252.97	11.199	0.926	2.39207
19	9	2	2	668.70	18.056	0.681	0.24244
20	9	2	5	827.16	18.084	0.820	0.46597
21	9	2	8	1045.83	16.612	1.043	1.13739
22	9	5	2	845.47	17.734	0.623	0.52127
23	9	5	5	987.35	15.512	0.716	1.09942
24	9	5	8	1198.88	12.589	0.894	2.12545
25	9	8	2	1083.54	18.150	0.886	1.15702
26	9	8	5	1183.48	14.077	0.934	2.08980
27	9	8	8	1324.12	9.3030	1.067	3.47044
	Obtained data for investigation of the accuracy of ANFIS models						
28	2	5	4	642.76	17.744	0.453	0.17338
29	3	3	6	699.85	19.699	0.618	0.18316
30	4	7	7	1021.06	13.991	0.751	1.40972
31	5	4	3	655.78	17.433	0.406	0.22285
32	6	7	7	1121.29	12.875	0.775	1.82230
33	5	3	7	833.51	18.521	0.712	0.53342

**Table 6:** The global error with different membership functions.

ANFIS Models	Membership Functions		
	gbellmf	gaussmf	gauss2mf
For energy consumption	0.24467	0.21472	0.31872
For expansion of the hole	0.23743	0.20364	0.25982
For material removal rate	0.22946	0.20043	0.24761

**Table 7:** The results for single optimization.

Scenarios	CAP	GAP	SV	ED (kJ)	HE (mm)	MRR (mm^3^/s)
For minimal energy consumed	9	8	8	10.111		
For minimal expansion	3	4	2		0.326	
For maximizing material removal rate	9	8	8			3.2822

**Table 8:** The pairwise matrix of the EDD responses.

EDD Responses	HE	MRR	ED
HE	1	1/3	1
MRR	3	1	5
ED	1	1/5	1

**Table 9:** The values of geometric mean and normalized weights.

EDD Responses	Geometric Mean	Normalized Weights
HE	0.6934	0.19
MRR	2.4660	0.66
ED	0.5848	0.15

**Table 10:** Setting parameters of the NCGA.

Parameter	Value/Function
Population size	20
Number of generations	40
Crossover type	1
Crossover rate	1
Mutation rate	0.01
Gene size	40

**Table 11:** Optimization results.

Method	Optimization Parameters			Responses		
	CAP	GAP	SV	ED (kJ)	HE (mm)	MRR (mm^3^/s)
Common values used	3	3	7	19.790	0.7112	0.29963
NCGA	6	5	4	16.470	0.5072	0.56845
Improvement				−3.32	−0.204	0.26882

**Table 12:** The results of confirmatory experiment.

Method	Optimization Parameters			Responses		
	CAP	GAP	SV	ED (kJ)	HE (mm)	MRR (mm^3^/s)
NCGA	6	5	4	16.470	0.5072	0.56845
Experiment	6	5	4	16.726	0.5204	0.57742
Errors (%)				1.55	2.60	1.58
